# Immunization coverage among refugee children in Berlin

**DOI:** 10.7189/jogh.09.010432

**Published:** 2019-06

**Authors:** Laila Fozouni, Christoph Weber, Andreas K Lindner, George W Rutherford

**Affiliations:** 1 School of Medicine, University of California, San Francisco, San Francisco, California, USA; 2 Department of Infectious Diseases, Vivantes Auguste – Viktoria Klinikum, Berlin and Airport Tempelhof Refugee Camp, Berlin, Germany

## Abstract

**Background:**

The Tempelhof refugee camp offers in-camp immunizations. Other camps, like Neukölln, rely on a centralized immunization system. We aimed to determine the impact of conflict on immunization rates of Syrian children and to measure the efficacy of in-camp immunization services.

**Methods:**

Families with children aged 1-5 in Tempelhof and Neukölln camps were surveyed. Surveys included siblings under the age of 18. Differences were compared using χ^2^ test.

**Results:**

Data on 179 children at Tempelhof and 40 children at Neukölln were collected. At Tempelhof, amongst Syrian children, 27.8% under the age of 5 were “fully immunized-memory,” in contrast to 73.7% over the age of 5 *(P =* 0.005). This difference in immunization rates by memory between the age groups was not observed in Afghani children (*P* = 0.34) or in Iraqi children (*P* = 0.10). Furthermore, compared to the 27.8% of Syrian children, 75% of Afghani children under the age of 5 were “fully vaccinated-memory” (*P* = 0.0009). Compared to Tempelhof, more children at Neukölln were partially immunized (93%) or had no immunizations (5%) (*P* < 0.001).

**Conclusion** These data suggest that conflict adversely affected immunization rates of Syrian children, and that offering in-camp immunization services may be a solution to increasing immunization rates.

In 2015, 890 000 refugees arrived in Germany [1], about 80 000 of them in Berlin [2]. The majority of refugees have come from Syria, Afghanistan, Iraq, and the Balkan region [3]. The mass influx of refugees in Germany and other host countries has created a major challenge for health care infrastructure [4]. Overcrowded and unsanitary living conditions can foster disease outbreaks. Measles, which is readily transmitted by air, is a particularly problematic for unimmunized refugee children. There were over 200 cases of measles in Jordan in 2013, and 1760 in Lebanon, up from nine just the year before [5]. Furthermore, in October of 2014 Berlin experienced an outbreak of measles, and as of August 2016, there had been over 1300 cases of measles. This outbreak affected the non-refugee population as well because of low population immunity, particularly in German adults [6].

Childhood immunization coverage is a sensitive indicator of the stability of health care that children receive, and high levels of immunization are needed to prevent transmission of many childhood vaccine-preventable diseases. For instance, for measles herd immunity is believed to require that 92%-94% of the population be immune [7]. Immunization programs in the countries from which refugees come have had mixed success. In Syria, before the conflict began in March 2011, health indicators were rising, including decreasing child mortality [5,8]. The war led to a rapid deterioration of Syria’s health care infrastructure and a gaping physician shortage [9]. A 2013 WHO study found that 40% of Syria’s ambulances had been destroyed by conflict, and that 57% of the public hospitals were markedly damaged [10]. It is estimated that of the 1.8 million Syrian children born since the start of the conflict, more than 50% remain unimmunized [11,12]. Based on WHO estimates, in 2010 Syria had a polio immunization rate (3 doses) of 83%, a measles immunization rate (2 doses) of 82%, and a tetanus immunization rate (3 doses) of 80%. In contrast, by 2017, these had fallen 53% for polio, 59% for measles and 48% for tetanus [13]. Syria experienced its first polio outbreak in over 15 years in 2013 and over 7000 cases of measles [5]. In contrast, in Afghanistan there has been an increase in immunization rates since 2001. In 2001, polio immunization coverage was 35%, measles immunization coverage was undocumented, and only 33% of children had been immunized with tetanus toxoid. By 2017, Afghanistan had a polio immunization rate of 60%, a measles immunization rate of 39% and a tetanus immunization rate of 65% [13]. This has been attributed to the mass influx of non-governmental organizations that were able to enter Afghanistan after the start of conflict and provide health care services [14]. In the past 20 years, Iraq has been impacted both by internal conflict as well as the conflict in neighboring Syria. In 2010 the Iraq had a polio immunization rate of 83%, a measles immunization rate of 91%, and a tetanus immunization rate of 84%. In 2017, this had fallen to coverage levels of 77%, 74% and 63%, respectively [13]. Moldova has experienced mass emigration due to economic and political instability. In 2010 polio coverage was 97%, measles coverage was 98%, and a tetanus coverage was 90%. By 2017, coverage had fallen to 90%, 92% and 88%, respectively [13]. Moldavan refugees arriving to Germany were primarily settled in camps in Berlin.

In Germany, childhood immunizations have historically been voluntary, though the Standing Committee on Vaccination (STIKO) makes national recommendations. Immunizations are documented on individual vaccination cards, and, as there is no national register documenting immunization coverage, school entrance examinations are the primary immunization data source used nationwide [15]. WHO estimates of German immunization rates as of 2017 were 94% for three polio doses, 93% for measles and 95% for tetanus [13].

In order to understand risk for outbreaks of vaccine-preventable childhood diseases, we conducted an immunization coverage survey in two refugee camps in Berlin.

## METHODS

This study had three aims: to understand the impact of conflict on immunization rates of Syrian refugee children, to understand risk factors for under-immunization among Syrian, Afghani, Moldavan, and Iraqi children living in refugee camps, and to estimate the impact of refugee camp immunization services on immunization rates of refugee children by comparing immunization card data from two camps in Berlin. For the purposes of this study, the immunizations assessed were polio, measles, and tetanus. The research was reviewed and approved by appropriate institutional ethical review committees both at UCSF and in Berlin.

### Setting

The two camps from which data was collected were the Tempelhof and Neukölln refugee camps. As of July 2016, the Tempelhof refugee camp housed about 1200 refugees and offered in-camp immunizations one day per week in the camp clinic [16]. The clinic did not actively monitor children’s immunization status but relied on families to return for immunizations. Clinic physicians also began approaching families to review immunization booklets and notify families of when they were due to return for immunization doses. The Neukölln camp had a population of about 500. It had not previously offered immunizations but had just begun immunization services during the week we conducted the survey there; during that week immunizations were given to four individuals.

Refugees typically receive several initial immunizations upon arrival in Berlin at centralized public health centers, such as the State Office for Health and Social Affairs (LAGESO). They are expected to receive future immunizations either by returning to a centralized public health center or from immunization events run by the centralized organizations at the camps. The refugee camps themselves generally do not offer immunization services in-house. Tempelhof refugee camp is unique in that it offers in-house immunizations run by the physicians in the Tempelhof medical clinic, and as such bypasses the centralized immunization system. This provides the opportunity to compare the efficacy of an in-house immunization campaign vs the centralized immunizations system. In Germany, immigrant children without immunization cards from their country of origin are generally considered unimmunized by authorities, and in order to be considered fully immunized, must restart their immunizations. Refugees are almost universally provided new, blank immunization cards upon arrival in Germany.

### Participants

The study population for those living in the Tempelhof and Neukölln camps were children in families that had one or more children between the ages of one and five; all children one year of age and older in these families were eligible. In the Tempelhof camp, we obtained a list of every family in the camp under the age of five. In Neukölln, we were not authorized to have a list of families and recruited participants during common meals. Five was chosen as the upper age limit to include only children born after the start of the Syrian conflict. One was chosen as a lower age limit because children under the age of one may be too young to receive immunizations of interest.

### Data collection

Each family was systematically approached, and consent was obtained. One author, a native Farsi speaker, communicated with Farsi speaking families directly. For Arabic and Russian speaking families, an official translator was used. At Tempelhof, a second round of interviews were conducted to capture families that arrived at the camp after the initiation of the first round of surveying. The first round of surveying included families at the camp before July, and the second round included families who arrived between July and August. In Neukölln we were only allowed to conduct interviews in common spaces. We went to the camp twice during dinner time and asked over loudspeaker in Farsi and Arabic for families with children under the age of 5 to bring immunization cards down to dinner. We then approached families and went through the same consent procedure. Parents at both camps that consented to participate in the study were asked a series of demographic questions regarding their country and region of origin, number of years of education, and past employment history. They were then asked questions about each of their children under the age of 18, regarding if they were born in hospital/home, the number of camps they had been to, duration of time since they had left their home countries and number of siblings. We then collected information regarding the immunization history of the children. Most families did not have immunization cards from their country of origin, and as such we organized data into two categories: *past immunization history* in country of origin based on parents’ memory, and *existing record* of immunizations provided by host countries and recorded on immunization cards issued by host country.

### Measures and analysis

For *past immunization history*, a child was classified as having “no immunizations,” being “partially immunized-memory” or being “fully immunized-memory,” with the understanding that each home country has a unique immunization schedule and recommended infant series. A child was classified as “fully immunized-memory” only if the parent remembered multiple immunization events, oral and intramuscular immunizations and felt certain that their child had received every available immunization required by their countries’ immunization schedule at their age group. If the child did not meet these criteria, but the parents remembered some immunizations, they were categorized as “partially immunized-memory.” For *existing immunization record*, we reviewed immunization cards issued in the host country and classified children as follows: “no immunizations,” “partially immunized,” “partially immunized but due to return,” and “fully immunized.” Those classified as “no immunizations” had no proof of immunizations since leaving their home country. Those classified as “partially immunized” had received some immunizations but were due for more and had no plan to return for more. Those classified as “partially immunized but due to return” had proof of partial immunization, but it was too soon for them to receive additional immunizations in their series and they had a set date for future immunizations. “Fully immunized” was defined as having all three polio, two measles, and three tetanus immunizations.

### Statistical methods

Differences between groups were compared using χ^2^ and Fisher exact test.

## RESULTS

There were 191 eligible children from 88 families in Tempelhof. At Neukölln, there were 28 families in total with children under the age of 5. Of the 88 families in Tempelhof, two did not consent to interviews and five were unable to be interviewed due to lack of Bosnian, Kurdish, Serbian, and Albanian translators, nine left the camp before being interviewed, one had not yet moved in and seven were never located. We interviewed parents from the 64 remaining families for data on 179 (94%) children. Of these, 78 (44%) were Afghani, 41 (23%) were Syrian, 23 (13%) were Iraqi, and 23 (13%) were Moldavan, with the remainder of children from other countries including Kuwait, Iran, and Turkmenistan. Ninety-two (51%) of these children were 1-5 years of age, and 87 (49%) were 5 years old or older. Of the 28 families in Neukölln, 12 families were interviewed; 40 children met our inclusion criteria. Of these children, 9 (23%) were Afghani, 22 (56%) were Syrian, 8 (20%) were Iraqi, none were Moldovan, and 1 (3%) was from Iran.

### Study population

Among all study participants, there were an average of 3.9 children per family. Mothers on average received 4.4 years of education and fathers on average received 4.7 years of education. Twenty-five percent of children in Tempelhof were born outside of the hospital setting (**Table 1**) compared to 58% of those in Neukölln (**Table 2**).

**Table 1:** Demographics at Tempelhof

		N (%)	By immunization status –memory		*P*-value
**Partially**	**Fully**
**Father employment**	**Worker**	118 (68)	42 (38)	70 (63)	*P* = 0.003
	**Business**	15 (9)	5 (33)	10 (67)	
	**Unemployed**	34 (20)	18 (69)	8 (31)	
	**Other**	7 (4)	0 (0)	6 (100)	
**Mother employment**	**Worker**	8 (4)	3 (38)	5 (63)	*P* = 0.33
	**Business**	3 (2)	0 (0)	3 (100)	
	**Unemployed**	160 (89)	64 (44)	81 (56)	
	**Other**	8 (4)	2 (25)	6 (75)	
Education* (in years)	**Father**	4.4	3.8	5	*P* = 0.06
	**Mother**	4.5	3.3	5.7	*P* = 0.0007
Number of siblings*		2.7	2.7	2.7	*P* = 0.78
**Born outside hospital**		44 (25)	19 (46)	22 (54)	*P* = 0.52

*Average number.

**Table 2:** Demographics at Neukölln

			Partially	Fully	
**Father employment**	**Worker**	23 (58)	5 (25)	15 (75)	*P* = 0.004
	**Military**	5 (13)	1 (10)	4 (90)	
	**Unemployed**	5 (13)	3 (60)	2 (40)	
	**Other**	7 (18)	7 (100)	0 (0)	
**Mother employment**	**Unemployed**	40 (100)	9 (30)	28 (70)	-
Education* (in years)	**Father**	6	2.5	6.9	*P* = 0.001
	**Mother**	3.8	1.4	4.5	*P* = 0.03
Number of siblings*		3.9	3.7	4.2	*P* = 0.55
**Born outside hospital**		23 (58)	6 (27)	16 (73)	*P* = 0.61

*Average number.

Children in both Tempelhof and Neukölln with fathers who were unemployed prior to migration were significantly less likely to be fully immunized by memory compared to children with employed fathers (*P* = 0.003, *P* = 0.004). There were no statistically significant differences observed for immunization rates by memory between children born inside vs outside hospitals. At Neukölln, both fathers and mothers of children who were fully immunized by memory had more years of education on average (*P* = 0.001, *P* = 0.03). At Tempelhof, this association was seen only among mothers (*P* = 0.0007). Finally, there were no statistically significant differences observed in immunization rates based on card data between hangars 1 and 2 of Tempelhof, where immunizations are given, and hangars 6 and 7, which are the farthest from the immunization clinic (*P* = 0.24).

### Immunization status at arrival in Germany

At the Tempelhof camp, based on parents’ history of immunization, 51% of all children under the age of 5 were fully immunized by memory compared to 68% of children over the age of 5 (*P* = 0.06). When looking specifically at Syrian children, 28% of children under the age of 5 were fully immunized by memory compared to 74% of children five years older and older (*P* = 0.005). This difference in immunization rates by memory between the age groups was not observed in Afghani (*P* = 0.34) or Iraqi children (*P* = 0.10 by Fisher exact test). Moreover, Syrian children less than five years of age were significantly less likely to be fully immunized than Afghani children less than five (28% vs 75%, *P* = 0.008) but not Iraqi children less than five (28% vs 53%, *P* = 0.13) (**Table 3**).

**Table 3:** Immunization status of refugee children according to parents’ recall by country of origin and age group, Tempelhof refugee camp, Germany, 2016

Country of origin	Age group	Unimmunized	Partially immunized	Fully immunized	Total
Syria	<5 y	0 (0)	13 (72)	5 (28)	18
	≥5 y	0 (0)	5 (26)	14 (74)	19
Afghanistan	<5 y	0 (0)	9 (25)	27 (75)	36
	≥5 y	0 (0)	14 (35)	26 (65)	40
Iraq	<5 y	0 (0)	7 (47)	8 (53)	15
	≥5 y	0 (0)	1 (13)	7 (88)	8
Moldova	<5 y	2* (22)	7 (78)	0 (0)	9
	≥5 y	0 (0)	3 (60)	2 (40)	5

*BCG only.

### Immunization status at time of study

Immunizations offered at Tempelhof included measles, mumps and rubella (MMR); varicella; tetanus and diphtheria toxoid and acellular pertussis (TdaP); inactivated polio vaccine (IPV); haemophilus influenza type b; hepatitits B; hepatitis A; rotavirus; quadrivalent meningococcal; seasonal influenza; and pneumococcal vaccines.

Based on review of immunization cards looking at measles, polio, and tetanus immunization rates, of the 179 children in Tempelhof, 83 (47%) were fully immunized or partially immunized and due to return, 92 (51%) were partially immunized, and 4 (2%) were unimmunized. There were no differences in immunization status among children under 5 years old and those 5 years old and older. However, children surveyed in the second round (ie, <2 months at the camp) were significantly more likely to have been unimmunized than those surveyed in the first round (*P* = 0.0016) (**Table 4**). In contrast, of the 40 children whose immunization cards were reviewed in Neukölln, 2 (5%) were unimmunized, 37 (92.5%) were partially immunized, none were partially immunized and due to return, and 1 (2.5%) was fully immunized. Compared to children in Tempelhof under five years of age, children in Neukölln were significantly less likely to be partially immunized and due to return or fully immunized (*P* < 0.001).

**Table 4:** Immunization status of refugee children as confirmed by immunization card by camp, Germany, 2016

Camp/round	Age group	Unimmun-ized	Partially immunized	Partially immunized due to return	Fully immunized*	Total
		**N (%)**	**N (%)**	**N (%)**	**N (%)**	**N**
Tempelhof –Round 1	<5 y	1 (1)	41 (55)	24 (32)	8 (11)	74
	≥5 y	0 (0)	45 (60)	26 (35)	4 (5)	75
	*Subtotal*	*1 (1)*	*86 (58)*	*50 (34)*	*12 (8)*	*149*
Tempelhof – Round 2	<5 y	1 (6)	5 (28)	12 (67)	0 (0)	18
	≥5 y	2 (17)	1 (8)	7 (58)	2 (17)	12
	*Subtotal*	*3 (10)*	*6 (20)*	*19 (63)*	*2 (7)*	*30*
Tempelhof – both rounds	<5 y	2 (2)	46 (50)	36 (39)	8 (9)	92
	≥5 y	2 (2)	46 (53)	33 (38)	6 (7)	87
	*Total*	*4 (2)*	*92 (51)*	*69 (39)*	*14 (8)*	*179*
Neukölln		2 (5)	37 (93)	0 (0)	1 (3)	40
Total		6 (3)	129 (59)	69 (32)	15 (7)	219

*Fully immunized defined as having all three polio, two measles, and three tetanus immunizations.

## DISCUSSION

Regarding our first aim, we found that refugee children in general have low rates of being fully immunized by both immunization card and history. Among Syrian children, immunization rates were significantly lower among those less than five years of age, who would have been born after 2011, suggesting a direct adverse effect of the Syrian civil conflict on child health care delivery. This difference was not seen among younger Afghani or Iraqi children where health care has been increasingly provided by non-governmental organizations. Indeed, Syrian children under the age of 5 had significantly lower immunization rates compared to Afghani children in the same age group. Similar figures of unimmunized Syrian children were found in an survey from Jordan and Lebanon, reflecting the general lack of immunization strategies within refugee camps and emphasizing the need for rapid immunization campaigns [17].

Because relying on caregiver recall for immunization history may not be accurate and reflect true immunization history, we endorse the current German practice to reissue home-based records to families reflecting the immunizations their children have received in Germany.

We also found that risk factors for under-immunization included lower education levels of parents as well as unemployment of the father. We did not find that number of siblings, education level of mother, or birth location contributed as significant risk factors.

Regarding our third aim, we also found that children in the Tempelhof camp, with established immunization services, were more likely to be partially immunized with scheduled catch-up immunization appointments or fully immunized than children living in the Neukölln camp where immunization services had just started at the time of the survey. Moreover, children who recently arrived in Tempelhof, sampled in the second survey round, were more likely to be completely unimmunized by record than those sampled in the first round. One possible reason is that these children were more recently displaced from home and had had less time to begin their immunizations in Germany. Given that half of these children had never been to another camp before, this is possible.

This study has some limitations. In Neukölln we were unable to interview parents of all children under five and instead had to rely on families approaching the interviewer. This may have created selection bias if families that had not received immunizations self-selected themselves to be interviewed or, conversely, tried to hide their lack of immunizations. Similarly, although to a lesser degree, our inability to locate and interview some families at Tempelhof may have led to selection bias. Moreover, we recognize that many families lost their children’s immunization cards during their flight to Europe, and the more recent immunization cards represent efforts at re-immunization. However, the low levels of immunization described by parents, especially among younger Syrian children, suggests a true immunization gap.

Despite these limitations, findings of low immunization rates both by immunization card and parents’ history are quite similar and distinctly lower from immunization rates in children in the European Union in general [18]. We also note that mandatory childhood immunization policies differ among countries in Europe and that immunization coverage in the general pediatric population is not ideal, creating a situation in which there is the potential not only for spread within camps but also from the camps to the general population [19]. Given the conditions in the camps and the poor immunization coverage we observed among younger children in these camps we conclude this may pose a threat of an outbreak of vaccine preventable disease first of all to the health of children living in the camps and less likely to the public health.

This study highlights several areas of possible intervention to improve rates of immunization for refugee children entering the EU. First, this study demonstrated a disparity in past immunization rates of children, suggesting that special attention should be paid towards populations at higher risk of being under-immunized, such as Syrian children. This study also highlights that children coming from areas with higher unemployment and lower education were at higher risk of under-immunization in their home countries, suggesting a demographic profile that should trigger additional attention. Finally, the finding that children in Tempelhof, with established immunization services, had significantly higher rates of immunization suggests that on-site clinics may be a solution.

## CONCLUSIONS

Our study provides quantitative data regarding immunization rates of Syrian children born since the start of conflict. The data can be used to support WHO estimates and identifies Syrian refugee children born after start of conflict as the most vulnerable to vaccine preventable disease. This information can guide immunization strategy. Furthermore, our study provides important data regarding the impact of an immunization campaign strategy unique to Tempelhof refugee camp, compared to the standard centralized immunization strategy and can be used to support in-camp immunizations as a public health intervention.
